# Biological Activity of *Artemisia mexicana*: Antimalarial, Immunomodulatory, and Antioxidant Effects in Experimental Malaria

**DOI:** 10.3390/ijms27177637

**Published:** 2026-08-26

**Authors:** Monserrat Sofía López-Padilla, Luis Antonio Cervantes-Candelas, Jesús Aguilar-Castro, Teresita de Jesús Nolasco-Pérez, Fidel Orlando Buendía-González, José Guillermo Avila-Acevedo, Edgar Antonio Estrella-Parra, Martha Legorreta-Herrera

**Affiliations:** 1 Posgrado en Ciencias Biológicas, Universidad Nacional Autónoma de México (UNAM), Unidad de Posgrado, Edificio D, 1° Piso, Circuito de Posgrados, Ciudad Universitaria, Coyoacán, Ciudad de México 04510, Mexico; yasared@comunidad.unam.mx (M.S.L.-P.); teresyta.qfb@gmail.com (T.d.J.N.-P.); 2 Laboratorio de Inmunología Molecular, Unidad de Investigación Química Computacional, Síntesis y Farmacología en Moléculas de Interés Biológico, División de Estudios de Posgrado e Investigación, Facultad de Estudios Superiores Zaragoza, Universidad Nacional Autónoma de México (UNAM), Iztapalapa, Ciudad de México 09230, Mexico; cervantescandelasluis@gmail.com (L.A.C.-C.); jesus.aguilar@zaragoza.unam.mx (J.A.-C.); fidelbuendia2@gmail.com (F.O.B.-G.); 3 Laboratorio de Fitoquímica, UBIPRO, Facultad de Estudios Superiores Iztacala, Universidad Nacional Autónoma de México (UNAM), Tlalnepantla de Baz 54090, Estado de México, Mexico; tuncomaclovio2000@yahoo.com.mx (J.G.A.-A.); estreparr@iztacala.unam.mx (E.A.E.-P.)

**Keywords:** *Artemisia mexicana*, antimalarial activity, *Plasmodium berghei* ANKA, immunomodulation, oxidative stress, murine malaria

## Abstract

Malaria pathogenesis results from a complex interplay among parasite burden, inflammation, and oxidative stress, all of which contribute to disease severity. This study evaluated the antimalarial, immunomodulatory, and antioxidant effects of a suspension of powdered aerial parts of *Artemisia mexicana* (*A. mexicana*) suspended in 0.5% carboxymethylcellulose (CMC), using a murine model of cerebral malaria. Male CBA/Ca mice were infected with *Plasmodium berghei* ANKA and treated with *A. mexicana.* Disease progression was evaluated through parasitemia, haemoglobin levels, body weight and temperature. Immunomodulatory effects were assessed by analysing splenic immune cell populations and pro- and anti-inflammatory cytokines, while antioxidant activity was determined in brain and spleen tissues. Treatment reduced parasitemia in a dose-dependent manner, with the highest efficacy observed at 1600 mg/kg, and also prevented haemoglobin loss and body weight decline. *A. mexicana* preserved CD4^+^ (T-helper) and CD8^+^ (T-cytotoxic) T-cell populations and modulated cytokine responses, increasing IFN-γ, IL-17, and IL-10 levels. Additionally, treatment increased glutathione peroxidase activity in the brain and spleen without affecting lipid peroxidation. These findings demonstrate that *A. mexicana* exerts antimalarial and immunomodulatory effects and modulates antioxidant responses, suggesting that the regulation of inflammation and redox balance contributes to improved outcomes during malaria infection.

## 1. Introduction

Malaria is the deadliest parasitic disease in the world and is caused by protozoa of the genus *Plasmodium* [1]. *Plasmodium falciparum* is responsible for most malaria-related deaths; infections manifest as fever, severe anaemia, neurological complications, and multiorgan dysfunction [2].

Effective control of *Plasmodium* infection relies on the activation of the host immune system through a coordinated inflammatory response. However, excessive or sustained inflammation contributes to immunopathology and tissue damage [3]. Persistent production of proinflammatory cytokines and activation of immune cells promote the generation of reactive oxygen species (ROS), thereby linking inflammation to oxidative stress during malaria infection [4,5]. Oxidative stress acts as a double-edged sword; although ROS are essential effector molecules used by the host to restrict parasite growth, their excessive accumulation causes oxidative damage, contributing to severe anaemia, endothelial dysfunction, and vascular damage [4,6]. Conversely, *Plasmodium* relies on a highly specialised antioxidant defence system to withstand the oxidative stress generated during haemoglobin digestion. This dependence creates a fragile redox balance that can be exploited by endoperoxide-containing compounds, such as artemisinin and its derivatives, which induce lethal oxidative damage within the parasite [7]. Excessive ROS also promote lipid peroxidation, generating products such as malondialdehyde (MDA) and 4-hydroxynonenal (4-HNE), which contribute to endothelial dysfunction, blood–brain barrier disruption, and tissue injury during cerebral malaria [5,8]. Therefore, therapies that simultaneously reduce parasite burden and mitigate inflammatory and oxidative damage represent promising approaches for improving clinical outcomes in malaria patients.

In traditional Chinese medicine, the genus *Artemisia*, particularly *Artemisia annua*, commonly known as Quinghao, has been used as an antimalarial since the 2nd century BC. However, it was not until 1971 that its main active compound, artemisinin, was isolated and used as an antimalarial treatment [9]. Although highly effective, the widespread use of artemisinin and artemisinin-based therapies has contributed to the emergence of resistant *Plasmodium falciparum* strains within less than three decades in several endemic regions. In this context, it has been shown that stable resistance to pure artemisinin developed after 16 serial passages, whereas complete resistance to whole-plant preparation was not achieved despite prolonged serial passaging [10]. These findings suggest that the complex phytochemical composition of plants may delay the development of resistance. These findings have encouraged the search for new therapeutic alternatives based on complex plant preparations which may interact synergistically [11].

Artemisinin, a sesquiterpene lactone derived from *Artemisia annua*, has multiple biological activities, including the induction of apoptosis, inhibition of enzymatic activity, and disruption of cellular metabolism [11]. Another species within this genus, *Artemisia mexicana* (syn. *Artemisia ludoviciana*), known as estafiate or hierba maestra, has been widely used in traditional Mexican medicine to treat fever, parasitic infections, inflammatory conditions, and metabolic disorders [12]. In vitro studies have demonstrated that its essential oil suppresses the expression of the proinflammatory cytokines interleukin 1β (IL-1β) and tumour necrosis factor alpha (TNF-α), as well as inflammatory mediators such as cyclooxygenase-2 (COX-2), inducible nitric oxide synthase (iNOS), and nuclear factor kappa-light-chain-enhancer of activated B cells (NF-κB), suggesting its potential anti-inflammatory properties [13]. Despite this evidence, the activity of *A. mexicana* as an antimalarial agent and immune-response modulator, as well as its ability to limit pathophysiological damage in malaria, has not been investigated experimentally. Given that immunopathology and oxidative stress are central to disease severity, assessing whether this plant has protective effects beyond parasite elimination, particularly by modulating inflammatory and redox responses, is of substantial interest.

Here, we evaluated the antimalarial, immunomodulatory, and antioxidant activities of a suspension of powdered aerial parts of *A. mexicana* plants in a murine model of cerebral malaria induced by *Plasmodium berghei* ANKA (*P. berghei* ANKA). Antimalarial efficacy was evaluated by monitoring parasitemia, and immunomodulatory effects were evaluated by analysing splenic immune cell populations and plasma levels of pro- and anti-inflammatory cytokines, as well as specific antibody levels. Additionally, oxidative stress was assessed by measuring superoxide dismutase (SOD), glutathione peroxidase (GPx) and catalase (CAT)-specific activities, alongside MDA and nitric oxide (NO) levels, to determine the potential protective effects of the plants. Collectively, these findings provide a basis for further investigation of the bioactive compounds present in *A. mexicana* and their potential development as adjunctive therapies against malaria.

## 2. Results

### 2.1. Detection and Quantification of Artemisinin in the Filtrate Obtained from the A. mexicana Suspension

**Figure 1:**
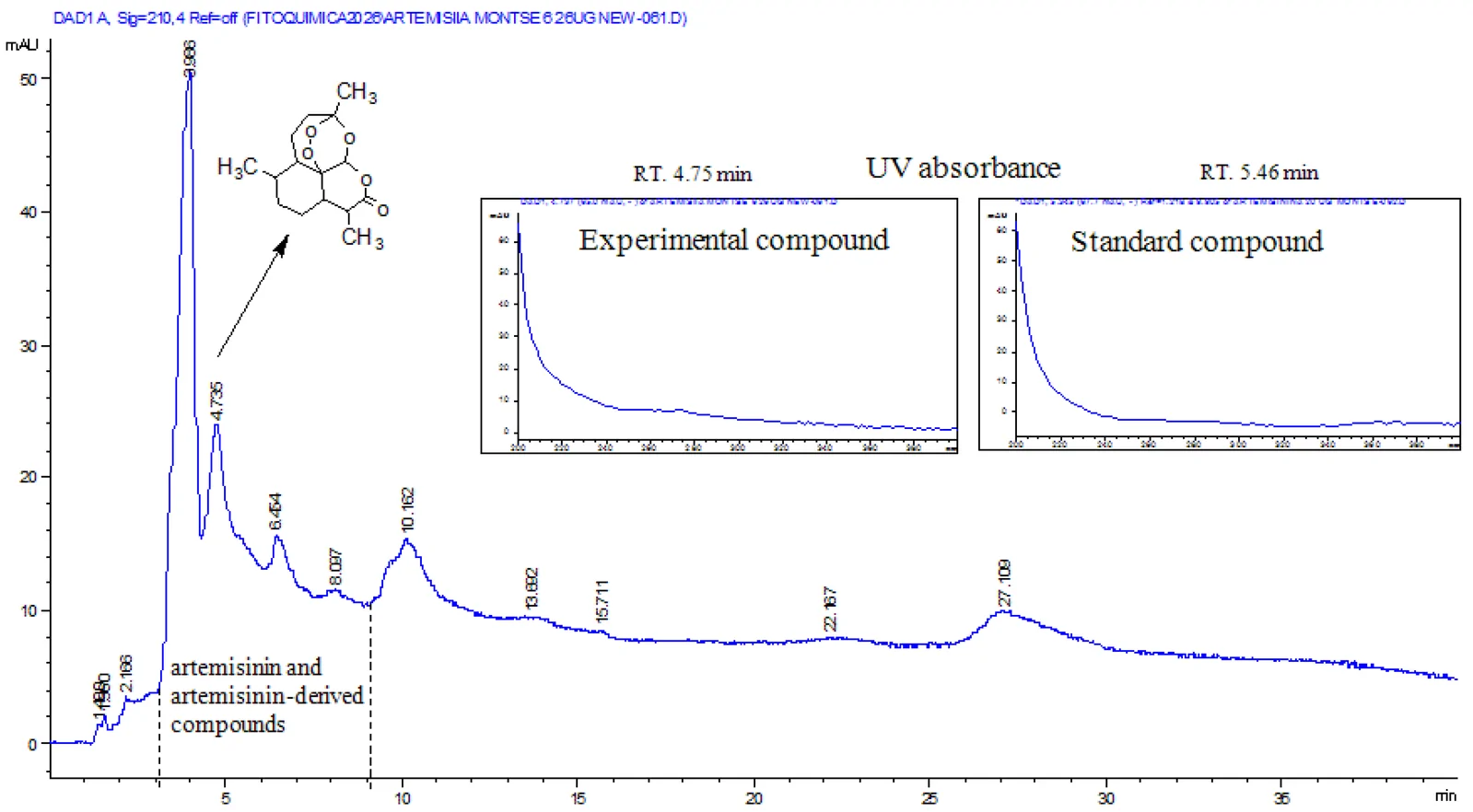
Chromatogram of the filtrate obtained from the *A. mexicana* suspension analysed by HPLC-DAD. Representative chromatographic profile of the filtrate obtained after vacuum filtration of the plant suspension. The arrow indicates the signal selected for comparison with the artemisinin standard. Insets show the UV absorption spectra corresponding to the experimental signal and the commercial standard.

The filtrate obtained from the plant suspension described in Section 4.1 was analysed by high-performance liquid chromatography with diode array detection (HPLC-DAD) to determine whether artemisinin was present. Quantitative analysis revealed an artemisinin concentration of 14.459 ± 1.669 mg per 1.5 g of dried aerial plant material (Figure 1).

The retention time of the commercial artemisinin standard was 5.46 min, whereas a signal with similar chromatographic characteristics was detected in the filtrate at 4.75 min. Additional peaks displaying UV absorption profiles comparable to those of the standard were observed between 3.98 and 9.0 min (Figure 1 and Figure S1). The chromatographic profile obtained from the filtrate is shown in Figure 1.

Following the chemical characterisation of the plant suspension, its antimalarial activity was evaluated in mice infected with *P. berghei* ANKA.

Although only artemisinin was experimentally confirmed by HPLC-DAD in the present study, several additional phytochemicals have previously been reported in *A. mexicana*. These compounds are summarised in Table 1 to provide a phytochemical context for the biological effects discussed below.

Only artemisinin was experimentally confirmed by HPLC-DAD in the present study. The remaining phytochemicals have been previously reported in *Artemisia ludoviciana* ssp. *mexicana* but were not experimentally identified in this work. They are included solely to provide phytochemical context for the discussion.

### 2.2. Determination of the Optimal Antimalarial Dose for A. mexicana

To determine the dose of *A. mexicana* with the greatest antimalarial activity, we assessed the effects of different concentrations of the plant on parasitemia in mice infected with *P. berghei* ANKA. The activity of the plant was compared with that of the positive control for antimalarial activity, chloroquine (CLQ), using a parasitemia suppression assay (Table 2).

**Figure 2:**
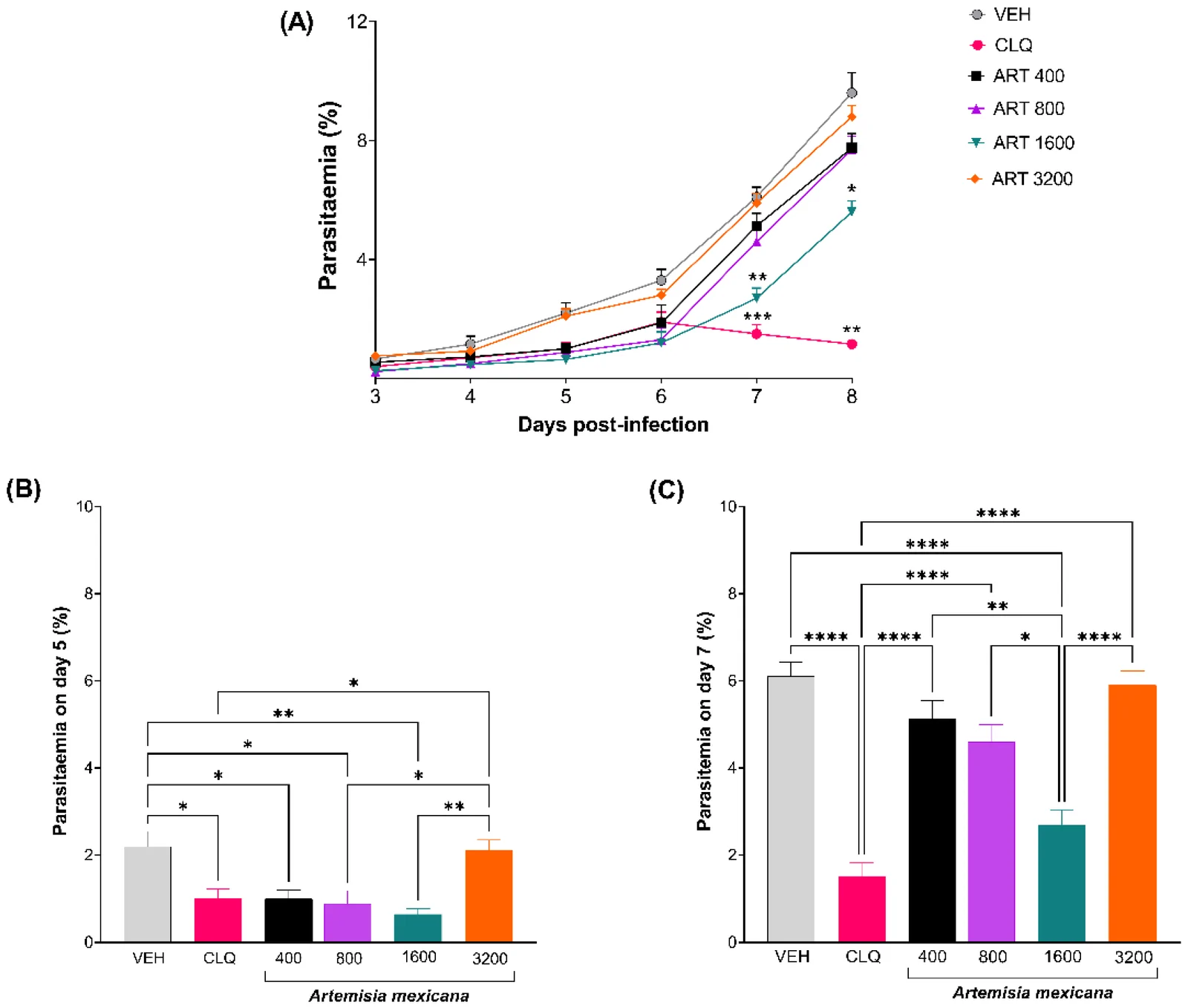
The dose of 1600 mg/kg *A. mexicana* resulted in the greatest antimalarial activity in CBA/Ca mice infected with *P. berghei* ANKA. Six groups of mice were infected with *P. berghei* ANKA and treated as follows: vehicle (VEH), chloroquine (CLQ), and the suspension of powdered aerial parts of *Artemisia mexicana* at doses of 400, 800, 1600 and 3200 mg/kg. Treatments were administered daily for four days. Parasitemia was assessed from day 3 post-infection. (**A**) Parasitemia kinetics; each point represents the geometric mean ± standard error of the mean (SEM) it was analysed using two ways ANOVA. The bars represent parasitemia on days 5 (**B**) and 7 (**C**) post-infection (n = 5). Significant differences were determined using one-way ANOVA, followed by the Bonferroni post hoc test. Significance levels are indicated as follows: *p* < 0.05 (*), *p* < 0.01 (**), *p* < 0.001 (***), and *p* < 0.0001 (****).

Treatment with *A. mexicana* reduced parasitemia in a dose-dependent manner starting on day 5 post-infection (Figure 2A,B). However, increasing the dose to 3200 mg/kg did not enhance antimalarial efficacy. In contrast, treatment with 1600 mg/kg produced the greatest antimalarial effect, achieving a 31.96% suppression of parasitemia by day 7 post-infection (Figure 2C and Table 2).

Based on these results, the 1600 mg/kg dose was selected for subsequent evaluation of the physiological and immunological effects of *A. mexicana*. To obtain preliminary safety information, uninfected mice treated with these doses (1600 and 3200 mg/kg) were monitored throughout the experimental period. No mortality or overt signs of acute toxicity were observed. In addition, gross macroscopic examination at necropsy revealed no visible abnormalities in the liver, spleen, kidneys, lung or brain compared with those in the untreated group.

### 2.3. Biochemical Assessment of the Hepatic and Renal Toxicity of A. mexicana

To further assess the potential toxicity of the 1600 mg/kg dose in uninfected mice, we measured biochemical markers of hepatic function, including total protein (TP), albumin (ALB), globulin (GLO), total bilirubin (TBIL), alanine aminotransferase (ALT), aspartate aminotransferase (AST), and gamma-glutamyl transferase (GGT), as well as markers of renal function, including urea and creatinine. No statistically significant differences were detected between the vehicle- and *A. mexicana*-treated groups for any quantifiable parameter (*p* > 0.05). In addition, ALT, GGT, and creatinine concentrations were at or below the lower limit of quantification in both groups. Overall, these findings provide no detectable biochemical evidence of hepatic or renal toxicity associated with *A. mexicana* treatment under the experimental conditions evaluated (Table 3).

### 2.4. Effects of A. mexicana on Physiological Parameters Associated with Experimental Malaria

**Figure 3:**
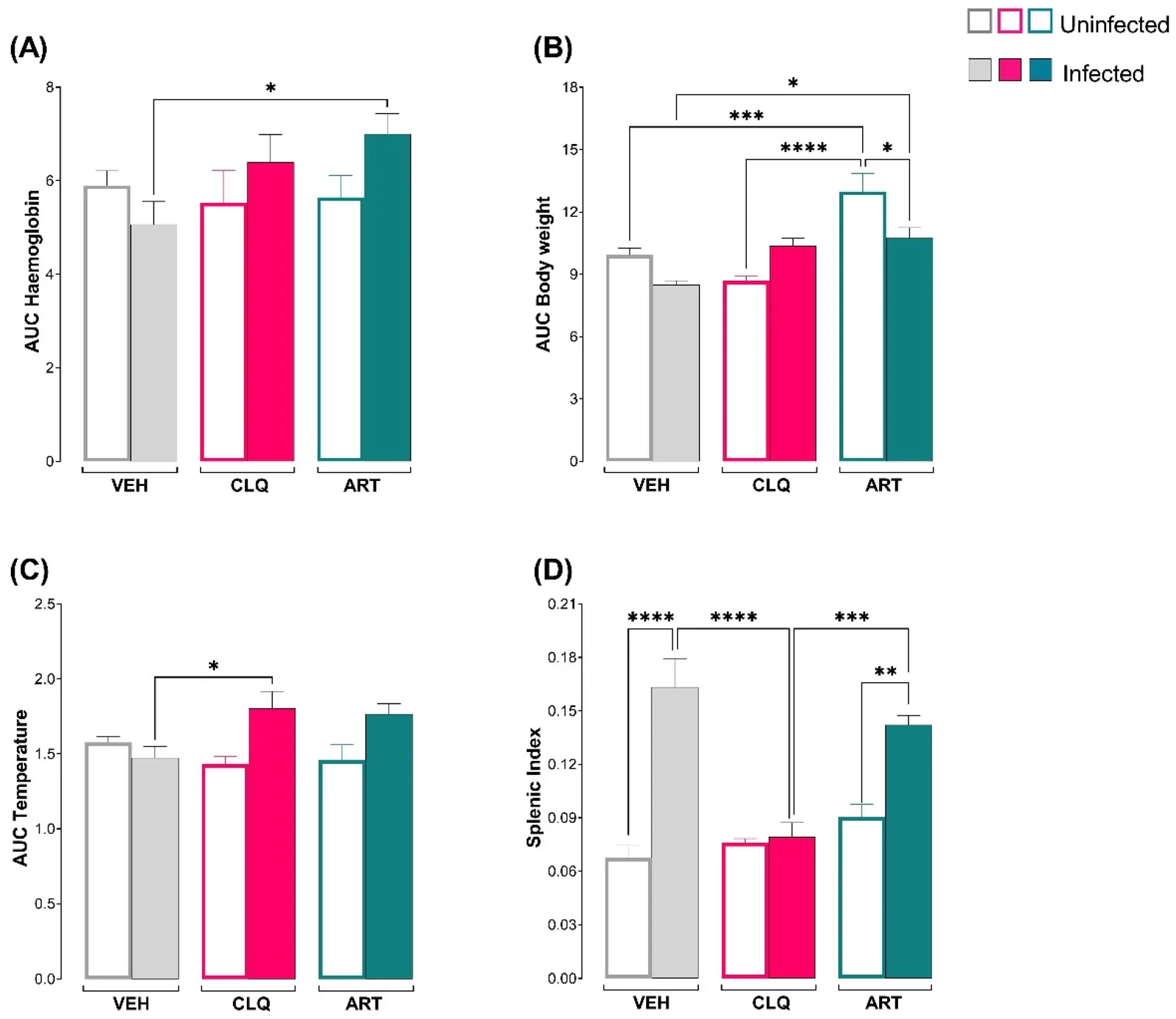
*A. mexicana* increased the haemoglobin levels and body weight in mice infected with *P. berghei* ANKA. Two groups of mice were treated with vehicle (VEH), two with chloroquine (CLQ) as a positive control for antimalarial activity, and two with the suspension of powdered aerial parts of *A. mexicana* (ART); the treatments were administered for 7 days starting on the day of infection. Only one group in each treatment was infected on day 0. Haemoglobin concentration (**A**), body weight (**B**), and temperature (**C**) were assessed daily. The area under the curve (AUC) was calculated for each parameter from day 0 to day 8 post-infection, and the splenic index (**D**) was calculated on day 8 post-infection (n = 5). Significant differences were determined using ANOVA followed by Bonferroni post hoc test. The empty bars represent the uninfected groups, whereas the coloured bars indicate the infected groups and represent the mean ± SEM. Significance levels are indicated as *p* < 0.05 (*), *p* < 0.01 (**), *p* < 0.001 (***) and *p* < 0.0001 (****).

Once the dose with the highest antimalarial activity had been determined, we assessed whether that dose of *A. mexicana* prevented complications associated with the disease, such as anaemia, weight loss, and hypothermia [24]. To this end, the haemoglobin concentration, body weight, and temperature were measured daily. Compared with vehicle treatment, *A. mexicana* treatment significantly increased the haemoglobin concentration and body weight in infected mice (Figure 3A,B). In contrast, no significant changes in body temperature were detected between the infected group treated with the suspension of powdered aerial parts suspension of *A. mexicana* and the group treated with vehicle (Figure 3C). The splenic index increased only in the vehicle-treated and *A. mexicana* aerial-part suspension-treated groups (Figure 3D).

### 2.5. Effects of A. mexicana on Splenic Immune Cell Populations During Experimental Malaria

**Figure 4:**
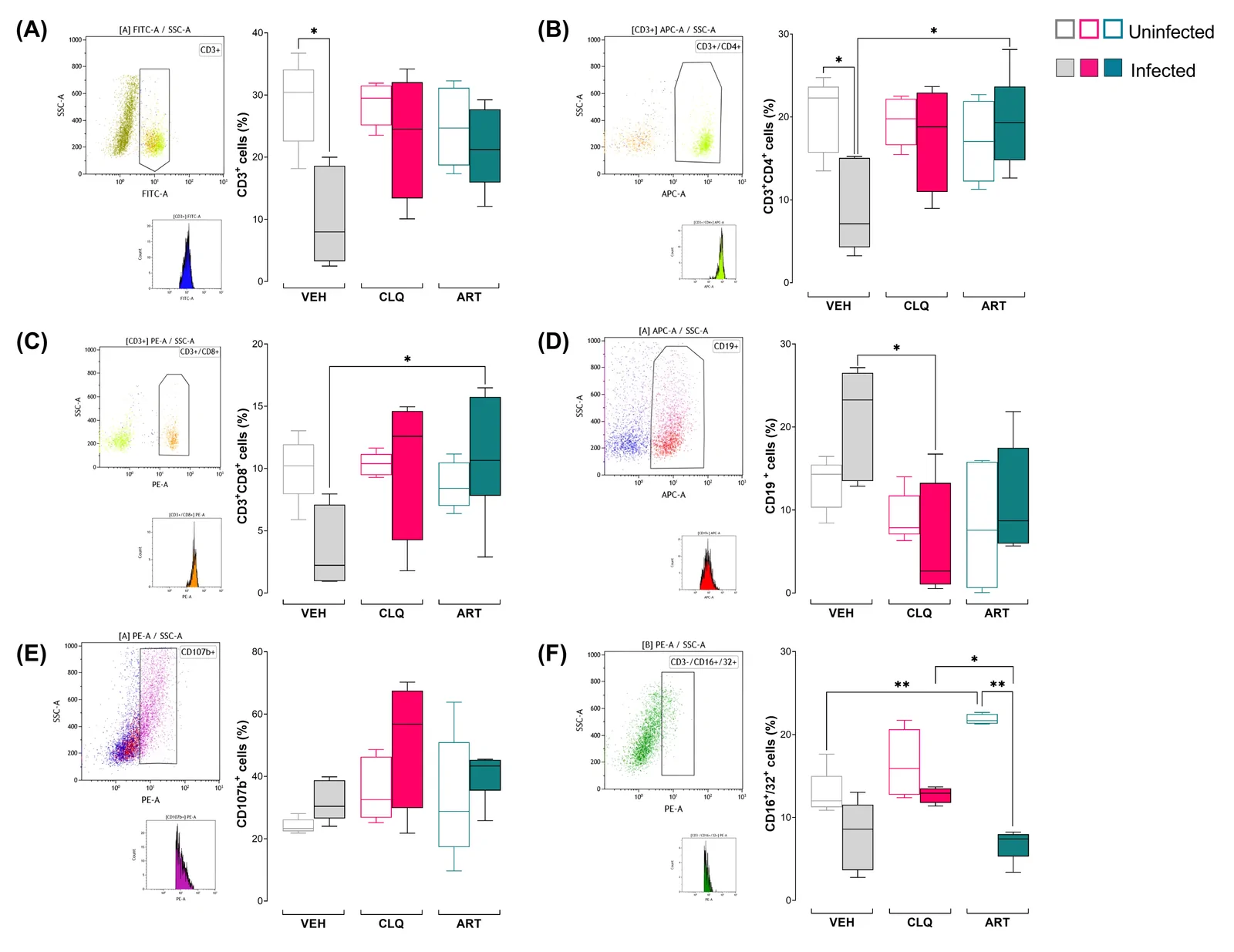
*A. mexicana* mitigated the decrease in helper and cytotoxic T lymphocytes in the spleens of mice infected with *P. berghei* ANKA. Two groups of male CBA/Ca mice (n = 5) were treated with vehicle (VEH), two with chloroquine (CLQ), and two with the suspension of powdered aerial parts of *A. mexicana* (ART), the treatments were administered for 7 days starting on the day of infection. Only one group in each treatment group was infected on day 0. On day 8 post-infection, the animals were euthanised, and the spleens were harvested for cellular immune characterisation. Flow cytometry analysed a total of 10,000 events per sample. The first panel shows a representative example of each cell population selection strategy, which was identified by the fluorescence emitted by fluorochrome-conjugated antibodies specific to each population and by cellular complexity. The bottom-left panel is the overlay showing the histogram for each population. The graphs on the right display the relative frequency of the following populations: total T lymphocytes (CD3^+^) (**A**), helper T lymphocytes (CD3^+^CD4^+^) (**B**), cytotoxic T lymphocytes (CD3^+^CD8^+^) (**C**), B lymphocytes (CD19^+^) (**D**), macrophages (CD107b^+^) (**E**) and NK cells (CD16^+^/CD32^+^) (**F**). The empty bars represent the uninfected groups, whereas the coloured bars indicate the infected groups and represent the mean ± SEM. The lines on the graphs indicate significant differences between groups, as determined by one-way ANOVA and the Bonferroni post hoc test *p* < 0.05 (*), *p* < 0.01 (**).

To further assess the effects of *A. mexicana* on the cellular immune response, splenic immune cell populations were evaluated. Total T lymphocytes (CD3^+^), helper T lymphocytes (CD3^+^CD4^+^), cytotoxic T lymphocytes (CD3^+^CD8^+^), B lymphocytes (CD19^+^), macrophages (CD107b^+^), and NK cells (CD16^+^/32^+^) were analysed. The administration of the plant prevented the reduction in the frequency of CD3^+^CD4^+^ and CD3^+^CD8^+^ cells (Figure 4A–C) caused by infection. In contrast, the populations of B lymphocytes and macrophages did not significantly change in response to the treatment (Figure 4D,E). Furthermore, treatment with the plant increased the NK cell population in uninfected mice, whereas infection reduced it in infected mice (Figure 4F).

### 2.6. Effects of A. mexicana on the Plasma Levels of Pro- and Anti-Inflammatory Cytokines in Mice Infected with P. berghei ANKA

**Figure 5:**
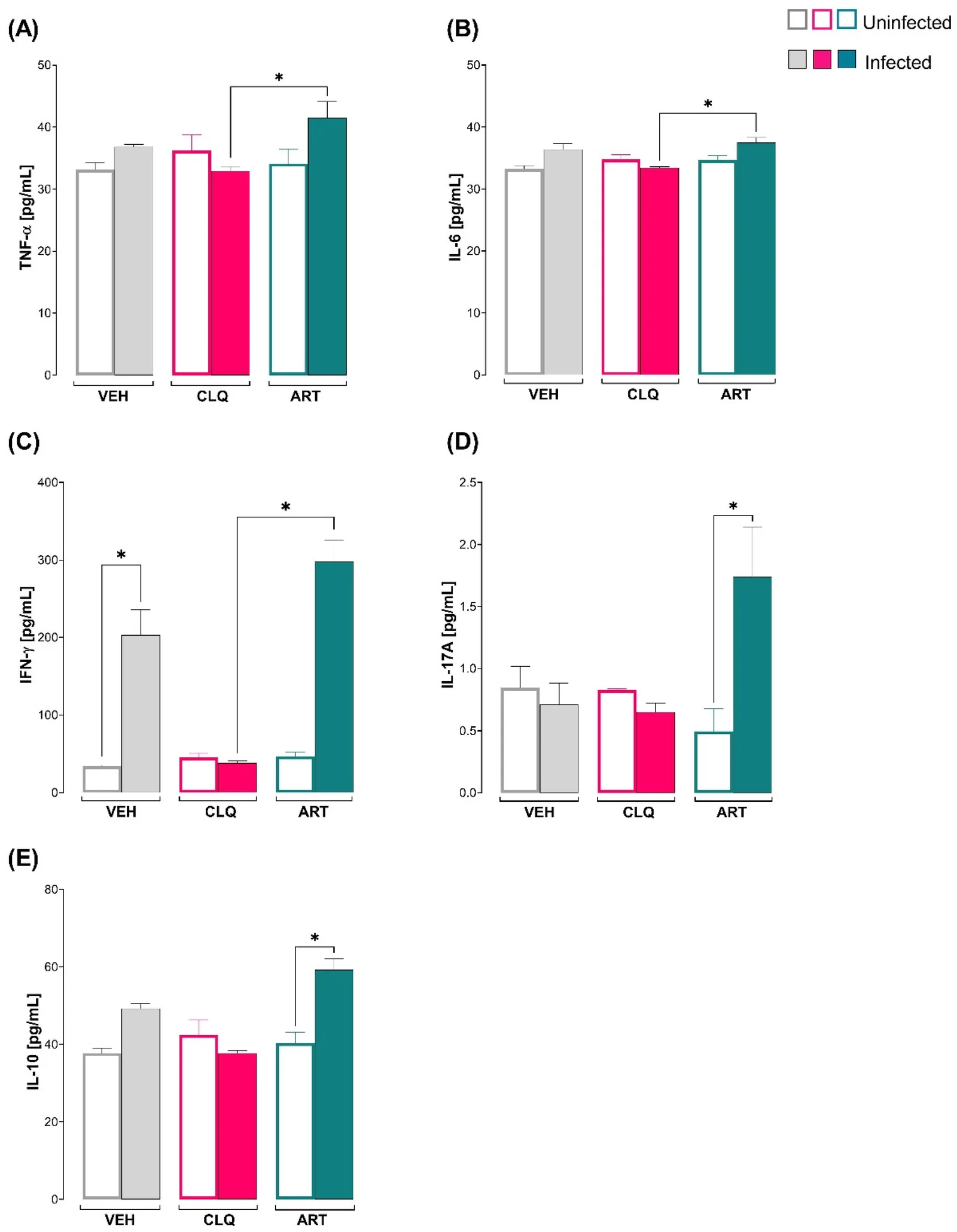
*A. mexicana* increased IFN-γ, IL-17A and IL-10 concentrations on day 8 post-infection in CBA/Ca mice infected with *P. berghei* ANKA. Two groups of male CBA/Ca mice (n = 5) were treated with vehicle (VEH), two with chloroquine (CLQ), and two with the suspension of powdered aerial parts of *A. mexicana* (ART), the treatments were administered for 7 days starting on the day of infection; only one group from each treatment was infected on day 0. On day 8 post-infection, the mice were euthanised, and plasma cytokine concentrations were determined by flow cytometry. The following cytokines were assessed: (**A**) tumour necrosis factor alpha (TNF-α), (**B**) interleukin 6 (IL-6), (**C**) interferon gamma (IFN-γ), (**D**) interleukin 17 (IL-17A) and (**E**) interleukin 10 (IL-10). The empty bars represent the uninfected groups, while the coloured bars indicate the infected groups and represent the mean ± SEM. Significant differences between groups were determined using one-way ANOVA, followed by the Bonferroni post hoc test, *p* < 0.05 (*).

To further investigate the effects of *A. mexicana* on the inflammatory immune response in *P. berghei*-infected mice, plasma cytokine concentrations were evaluated. To this end, the plasma concentrations of proinflammatory cytokines (TNF-α, IL-6, IFN-γ and IL-17A) and anti-inflammatory cytokines (IL-10) were determined in uninfected and infected mice treated with plants. The infection increased the concentration of IFN-γ in the vehicle-treated mice. However, compared with those in the CLQ and the antimalarial positive control groups, the concentrations of TNF-α, IL-6, IFN-γ and IL-17A increased in infected animals treated with the powdered aerial-part suspension of *A. mexicana* (Figure 5A–D). In addition, IL-10 was not significantly increased in infected vehicle-treated mice but was significantly elevated following *A. mexicana* treatment (Figure 5E).

### 2.7. Effects of A. mexicana on Plasma Antibody Levels in Mice Infected with P. berghei ANKA

**Figure 6:**
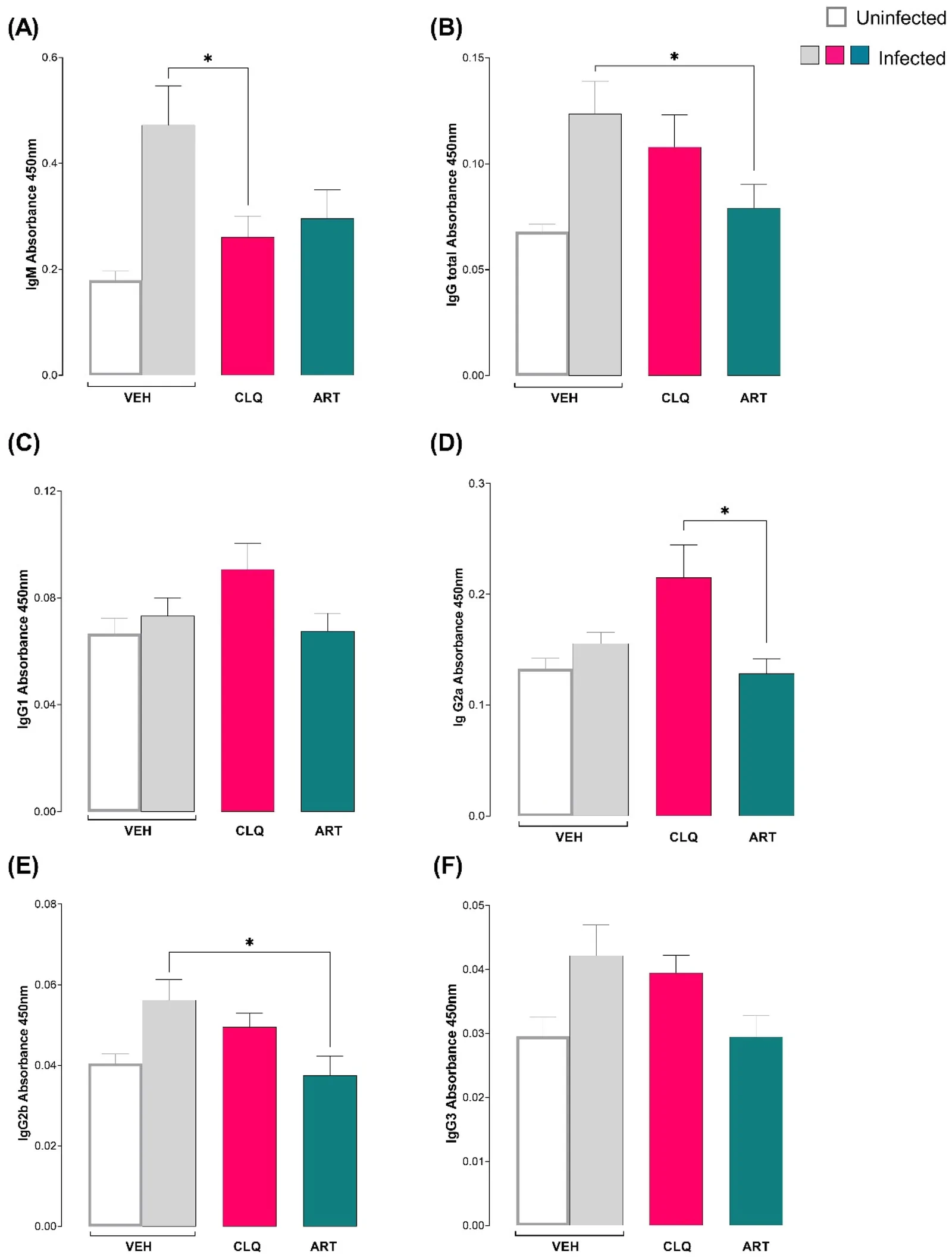
*A. mexicana* reduced total IgG and IgG2b levels in CBA/Ca mice infected with *P. berghei* ANKA. Three groups of male CBA/Ca mice (n = 5 per group) were treated with vehicle (VEH), chloroquine (CLQ), or *A. mexicana* (ART) for 7 days. Infection was performed on day 0, and on day 8 post-infection, the animals were euthanised to obtain blood samples. Plasma antibody levels were determined via ELISA. The levels of (**A**) IgM, (**B**) total IgG, (**C**) IgG1, (**D**) IgG2a, (**E**) IgG2b, and (**F**) IgG3 were assessed. The bars represent the mean ± SEM. The lines above the bars indicate significant differences between groups, which were calculated using one-way ANOVA followed by the Bonferroni post hoc test (*p* ≤ 0.05; *).

To further assess the effects of *A. mexicana* on the adaptive immune response, plasma antibody levels were evaluated. To this end, we compared total IgG and IgG subclasses, as well as IgM, in infected mice treated with the suspension of powdered aerial parts of the plant, with those in infected mice treated with vehicle. Treatment with *A. mexicana* did not affect IgM levels (Figure 6A); however, total IgG levels decreased in animals treated with *A. mexicana* compared with those in the group treated with vehicle (Figure 6B). When the IgG subclasses were analysed, no differences in IgG1 levels were observed between the groups treated with the plant and the vehicle (Figure 6C). In contrast, the IgG2a levels in the plant-treated group were lower than those in the CLQ-treated group (Figure 6D). Moreover, compared with those in the vehicle-treated group, the IgG2b levels in the plant-treated group decreased (Figure 6E). Finally, no changes were detected in the IgG3 subclass between the different groups (Figure 6F).

### 2.8. Effects of A. mexicana on Antioxidant Enzymes in the Brain and Spleen During Experimental Malaria

**Figure 7:**
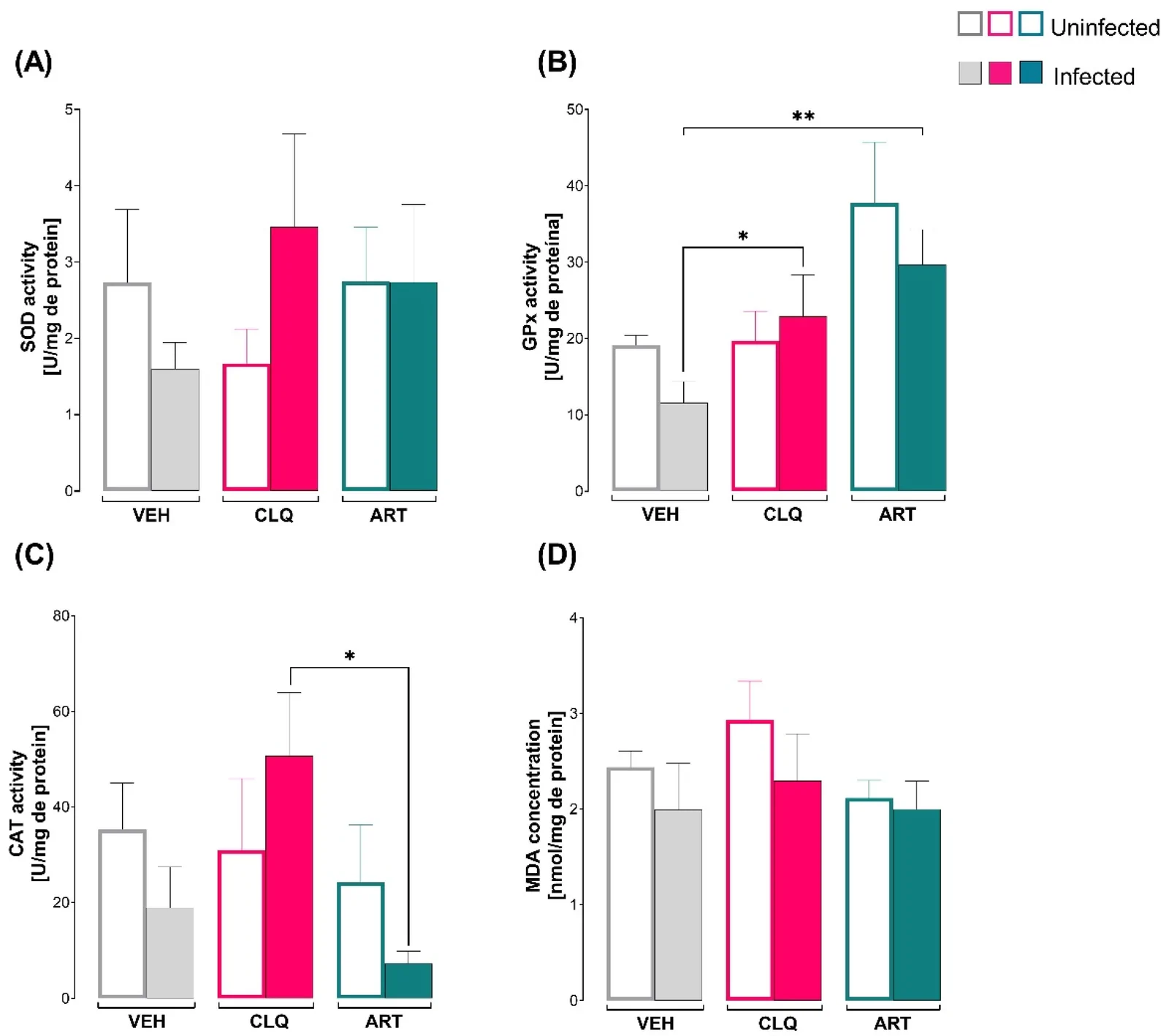
Effects of *A. mexicana* on the specific activity of superoxide dismutase (SOD), glutathione peroxidase (GPx), catalase (CAT), and MDA levels in the brains of mice infected with *P. berghei* ANKA. Two groups of male mice (n = 5) were treated with vehicle (VEH), two with chloroquine (CLQ) and two with the suspension of powdered aerial parts of *A. mexicana* (ART) for 7 days. Only one group from each treatment was infected on day 0, and on day 8 post-infection, the animals were euthanised to obtain brain samples. The activity of (**A**) superoxide dismutase (SOD), (**B**) glutathione peroxidase (GPx), and (**C**) catalase (CAT) and the concentration of (**D**) malondialdehyde (MDA) were assessed in the six groups. The empty bars represent the uninfected groups, whereas the coloured bars indicate the infected groups and represent the mean ± SEM. The lines above the bars indicate significant differences between groups, which were calculated using one-way ANOVA followed by the Bonferroni post hoc test *p* ≤ 0.05 (*), *p* ≤ 0.01 (**).

To further characterise the biological effects of *A. mexicana*, oxidative stress was assessed by measuring antioxidant enzyme activity and lipid peroxidation in the brain and spleen. Specifically, the activities of SOD, GPx, and CAT, as well as the levels of malondialdehyde (MDA), which is an indicator of lipid peroxidation, were determined. In the brain, no differences in SOD activity were observed between the groups (Figure 7A). In contrast, compared with that in the infected group treated with vehicle, GPx activity increased in the group treated with the suspension of powdered aerial parts of *A. mexicana* (Figure 7B). In addition, compared with the CLQ-treated mice, animals treated with *A. mexicana* exhibited decreased CAT activity (Figure 7C). However, no changes in the MDA concentration were detected in any of the groups evaluated (Figure 7D).

**Figure 8:**
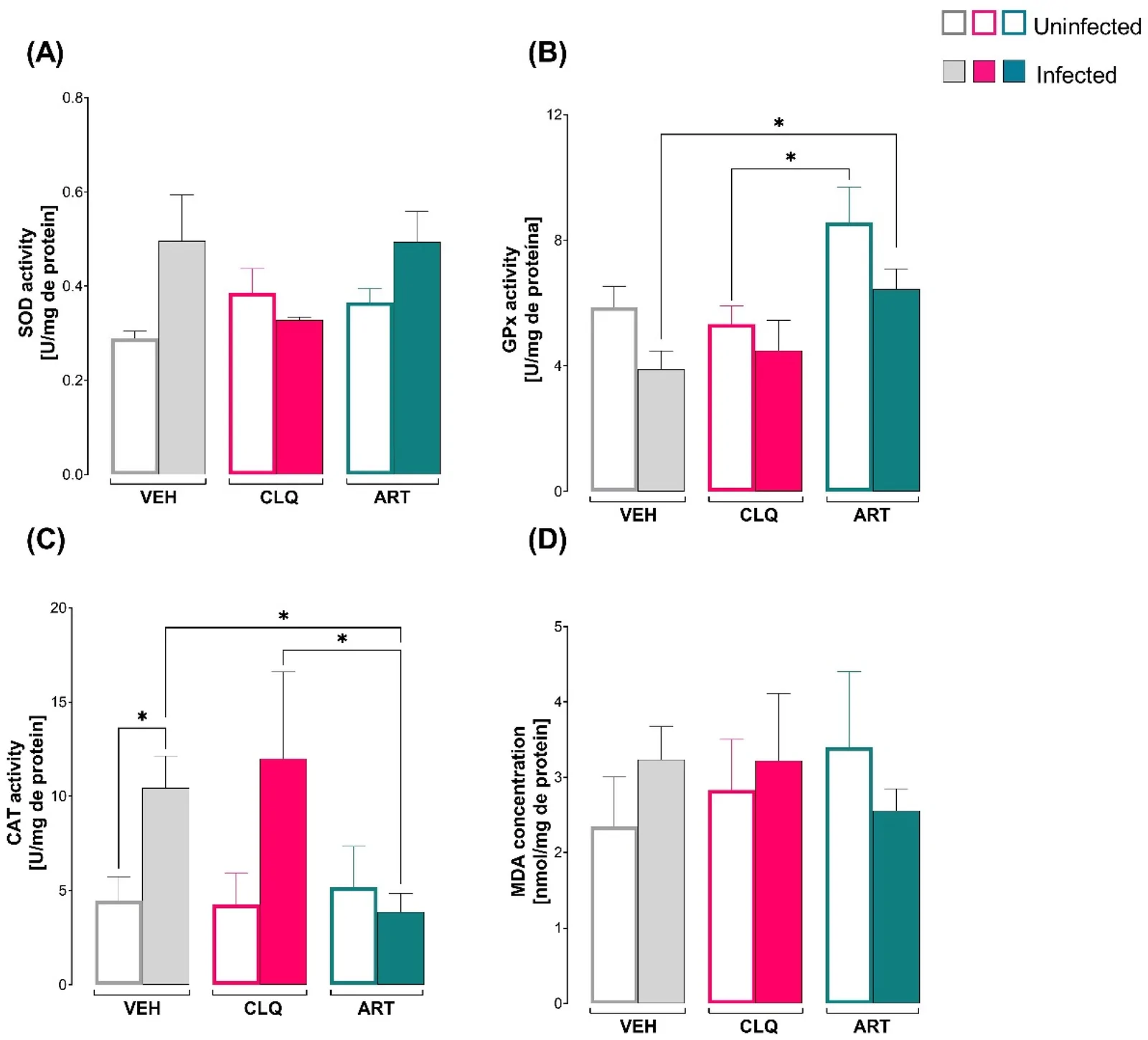
Effects of *A. mexicana* on the activity of SOD, GPx, CAT and MDA in the spleens of CBA/Ca mice infected with *P. berghei* ANKA. Two groups of male mice (n = 5) were treated with vehicle (VEH), two with chloroquine (CLQ), and two with *A. mexicana* (ART) for 7 days. Only one group from each treatment was infected on day 0. On day 8 post-infection, the animals were euthanised, and their spleens were collected. The activity of (**A**) superoxide dismutase (SOD), (**B**) glutathione peroxidase (GPx), and (**C**) catalase (CAT), and the concentration of (**D**) malondialdehyde (MDA) were assessed in the six groups. The empty bars represent the uninfected groups, while the coloured bars indicate the infected groups and represent the mean ± SEM. The lines above the bars indicate significant differences between groups, which were calculated using one-way ANOVA followed by the Bonferroni post hoc test (*p* ≤ 0.05 *).

Similarly, in the spleen, no significant differences in SOD activity were observed between the groups (Figure 8A). In contrast to that in the vehicle-treated group, GPx activity in the suspension of powdered aerial parts of *A. mexicana*-treated group increased (Figure 8B). Compared with the vehicle-treated group, the group of mice treated with *A. mexicana* presented decreased catalase activity (Figure 8C). However, no changes in the MDA concentration were detected between the groups evaluated (Figure 8D). Nitrite/nitrate (NOx) levels were additionally determined as an indirect indicator of nitric oxide production. However, no significant differences were observed in brain or spleen tissues following *A. mexicana* treatment (Supplementary Figure S2).

## 3. Discussion

Our findings indicate that *A. mexicana* possesses antimalarial and immunomodulatory activity by reducing parasitemia and preserving T-lymphocyte populations, selectively modulating the cytokine profile, and reducing the humoral response without increasing oxidative stress during infection with *P. berghei* ANKA.

Chemical characterisation of the filtrate obtained from the plant suspension confirmed the presence of artemisinin and additional chromatographic signals with UV absorption profiles similar to those of artemisinin. In the present study, HPLC-DAD analysis detected and quantified artemisinin in a suspension of powdered aerial parts of *A. mexicana*, yielding a concentration of 14.459 ± 1.669 mg per 1.5 g of dried aerial plant material. In addition, several chromatographic signals exhibiting UV absorption profiles similar to those of artemisinin were observed between 3.98 and 9.0 min, suggesting the presence of structurally related compounds, as reported in the literature [25]. Previous phytochemical studies of *A. mexicana* (*A. ludoviciana* ssp. *mexicana*) have reported the presence of sesquiterpene lactones, including estafiatin, achillin, ridentin, arglanin, ludovicin, A. armexifolin and armefolin, as well as flavonoids such as eupatilin and artemetin [19,25]. Although additional structural analyses would be required for definitive identification, some of the analytes detected in the present chromatographic profile may correspond to compounds previously reported for this species.

After confirming the presence of artemisinin in the suspension of powdered aerial parts of *A. mexicana,* we confirmed the establishment of the experimental cerebral malaria model by detecting *Plasmodium berghei* ANKA DNA in the brains of infected mice (Supplementary Figure S3).

Next, we evaluated the antimalarial activity of the suspension of powdered aerial parts of *A. mexicana* by quantifying parasitemia in *P. berghei* ANKA-infected mice treated with different concentrations of the plant. The dose of 1600 mg/kg, corresponding to 40 mg of powdered plant material administered to a 25 g/mouse, produced the greatest reduction in parasitemia, suggesting a dose-dependent effect; however, the administered dose (1600 mg/kg) represents the mass of powdered aerial plant material and should not be interpreted as an equivalent dose of purified artemisinin. However, this was not the highest evaluated dose, suggesting the presence of a nonlinear response, a phenomenon previously reported for complex plant extracts, in which interactions among bioactive metabolites may influence the bioavailability or biological activity of the active compounds [26,27].

The antimalarial activity observed may therefore result from the combined action of multiple bioactive metabolites present in the suspension of powdered aerial parts of the plant. Artemisinin and other sesquiterpene lactones belong to the terpenoid family, and these compounds are known to interfere with essential metabolic pathways in *Plasmodium*, including isoprenoid biosynthesis, which is required for parasite growth and survival [28]. Similarly, flavonoids reported in *Artemisia* species are associated with antioxidant and immunomodulatory activities [29], which may complement the antiparasitic effects of sesquiterpene lactones. Therefore, the biological activity observed in the present study is likely attributable to the phytochemical complexity of the suspension of powdered aerial plant suspension rather than to a single constituent. In addition, the antimalarial activity observed could be associated with metabolites reported in *A. mexicana*, particularly sesquiterpene lactones such as estafiatine or aquiline [28,30,31]. These compounds belong to the terpenoid group, a family widely recognised for their biological activity. Several terpenoids can interfere with the biosynthesis of dolichol and ubiquinones in *Plasmodium* by inhibiting the isoprenoid pathway, which is essential for parasite growth and survival [28]. In this context, the sesquiterpene lactones present in *A. mexicana* may contribute to the observed antimalarial activity through similar metabolic mechanisms. However, this relationship should be considered a hypothesis supported by the literature rather than direct experimental evidence.

Based on these findings, the 1600 mg/kg dose resulted in the greatest parasite suppression on day 5, and parasitemia increased after completion of the four-day suppressive treatment, indicating that parasite growth resumed once treatment was discontinued. This finding is consistent with the purpose of the four-day suppressive test, which is designed to evaluate a candidate compound’s ability to inhibit parasite multiplication during treatment rather than achieve complete parasite clearance. For that reason, in the second experiment, the treatment was extended to 7 days.

In addition to its antiparasitic effect, treatment with the suspension of powdered aerial parts of *A. mexicana* improved the physiological parameters associated with the severity of experimental malaria. During *Plasmodium* infection, anaemia is a common complication resulting from the destruction of both infected and uninfected erythrocytes, mediated by both parasite replication and immune and inflammatory mechanisms, including haemolysis, splenic phagocytosis, and suppression of erythropoiesis [28,30]. In this study, the administration of the suspension of powdered aerial parts of *A. mexicana* helped maintain the haemoglobin concentration in infected mice, suggesting a protective effect against the development of anaemia. Some species of the genus *Artemisia* reduce hepcidin expression [31,32], a key hormone involved in the regulation of iron metabolism; its overexpression limits iron availability for erythropoiesis and contributes to inflammation-associated anaemia [32]. In an experimental model of osteoporosis, *Artemisia* extracts reduced serum hepcidin levels and increased iron bioavailability [31], suggesting a possible mechanism for the preservation of the haemoglobin concentration observed in this study. However, given that IL-6 is the central regulator of hepcidin and that in our model, the plant did not reduce this cytokine, the protective effect likely does not depend exclusively on this pathway. In contrast, the increase in IL-1 levels suggested that the activation of anti-inflammatory mechanisms can limit damage and promote erythropoietic stability.

Consistently, the administration of the suspension of powdered aerial parts of *A. mexicana* increased the body weight in infected mice compared with the infected mice treated with vehicle. The loss of weight in infected mice has been linked to the overproduction of proinflammatory cytokines, particularly tumour necrosis factor alpha (TNF-α) and gamma interferon (IFN-γ), which contribute to the development of cachexia [24,33]. Interestingly, in our study, the suspension of powdered aerial parts of the plant increased the levels of both cytokines and maintained body weight in the infected mice, suggesting that the plant may modulate the metabolic effects of inflammation.

Furthermore, weight gain was observed in uninfected mice treated with *A. mexicana*, suggesting a possible effect on basal energy metabolism. Similar results have been reported for other plant species, such as *Cymbopogon citratus*, where dietary supplementation increases body weight and improves nutrient utilisation efficiency in uninfected animals [34]. Taken together, these findings suggest that *A. mexicana* may influence energy balance by promoting better nutrient utilisation or by modulating the balance between anabolic and catabolic processes during infection.

In the spleen, the central organ of the immune response against *Plasmodium* [35], the administration of *A. mexicana* increased the frequency of CD3^+^CD4^+^ and CD3^+^CD8^+^ T lymphocytes during infection. Given that *Plasmodium* infection induces T-cell apoptosis due to immune activation [36,37], this finding may be related to previous observations showing that *A. mexicana Artemisia* species reduce the depletion of the CD4^+^ population following intense activation [38]. These findings are important for cerebral malaria, as T-cell depletion is associated with disease progression and increased mortality.

On the other hand, although B lymphocytes in mice treated with the plant tended to decrease, no changes were observed in IgM levels, whereas total IgG and IgG2b levels decreased. This pattern suggests that the plant inhibits the proliferation and activation of B cells.

In malaria, the IgG2b subclass is involved in effector mechanisms such as opsonisation and complement activation, which contribute to parasite elimination but can also exacerbate tissue damage when produced in excess [39]. In this context, the decrease in total IgG and IgG2b, and the decrease in B lymphocyte frequency, suggest that the suspension of powdered aerial parts of *A. mexicana* may influence B-cell function rather than cell frequency.

Regarding to natural killer (NK) cells, the frequency of these cells increased in uninfected mice treated with the plant, whereas in infected animals, it decreased, accompanied by an increase in IFN-γ. Similar immunomodulatory effects of plant-derived compounds on natural killer cell function have been reported, including enhanced NK activity following exposure to herbal extracts of *Echinacea* and ginseng [40]. This pattern suggests that the suspension of powdered aerial parts of *A. mexicana* exert an immunomodulatory effect dependent on the host’s physiological state, stimulating immune activity under basal conditions and preventing cellular exhaustion during infection, without compromising the production of cytokines necessary for parasite control.

Consistent with these immunomodulatory effects, *A. mexicana* increased the levels of IL-10, an anti-inflammatory cytokine that plays a key role in limiting immunopathology in malaria [41]. IL-10 contributes to controlling parasite-induced inflammation while preventing excessive immune responses, which may explain the stability of lymphocyte populations and the preservation of physiological parameters in the treated mice.

Furthermore, the increase in IFN-γ and IL-17 levels suggests coordinated activation of T helper 1 cell (Th1) and Th17 responses, both of which contribute to controlling parasitic growth [42]. However, excessive production of these cytokines has also been implicated in the immunopathology of cerebral malaria [43,44]. In the present study, these increases occurred together with elevated IL-10 levels, preservation of CD4^+^ and CD8^+^ T-cell populations, reduced parasitemia, and improved physiological parameters. This pattern suggests a regulated, rather than exacerbated, inflammatory response, in which IL-10 may help limit excessive inflammation while preserving effective antiparasitic immunity.

Finally, oxidative stress is closely linked to the inflammatory response in cerebral malaria, as excessive ROS production contributes to endothelial dysfunction and neurological damage [4]. Because ROS promote lipid peroxidation, MDA and NO were evaluated as markers of oxidative damage, along with antioxidant enzyme activity. Treatment with the suspension of powdered aerial parts of *A. mexicana* increased GPx activity in both the brain and the spleen, without altering the MDA or NO concentrations. Since GPx constitutes one of the main enzymatic systems responsible for hydrogen peroxide detoxification [45], its increased activity may have been sufficient to maintain peroxide homeostasis and limit lipid peroxidation under the experimental conditions. In contrast, the higher CAT activity observed in the VEH and CLQ groups may represent a compensatory response to increased hydrogen peroxide accumulation and oxidative stress.

Collectively, these findings suggest that *A. mexicana* enhances endogenous antioxidant defences while preserving redox homeostasis, potentially through the coordinated action of GPx and other antioxidant phytochemicals present in the powdered aerial plant suspension.

**Figure 9:**
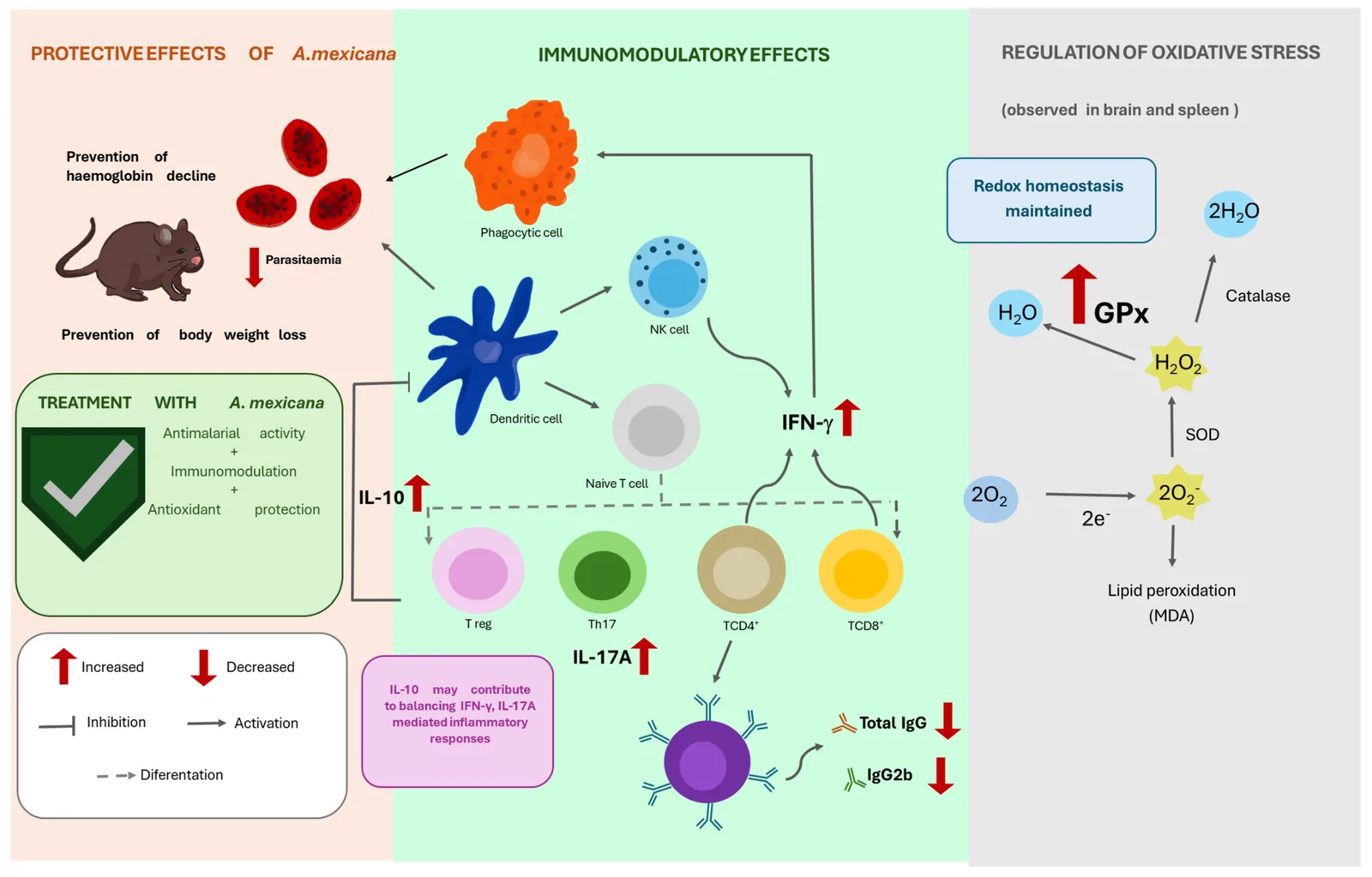
Proposed mechanism underlying the protective effects of *A. mexicana* during experimental *Plasmodium berghei* ANKA infection. The diagram summarises the experimental findings of the present study. Treatment with the suspension of powdered aerial parts of *A. mexicana* reduced parasitemia while preserving haemoglobin levels and body weight. It also maintained splenic CD4^+^ and CD8^+^ T-cell populations and increased IL-10, IFN-γ, and IL-17A levels. The concomitant increase in IL-10 may help regulate IFN-γ- and IL-17A-mediated inflammatory responses, thereby promoting a balanced immune response during infection. Antioxidant modulation was evidenced by increased glutathione peroxidase (GPx) activity in the brain and spleen, with no change in malondialdehyde (MDA) levels, which is consistent with the maintenance of redox homeostasis. Collectively, these findings support a proposed mechanism whereby the protective effects of *A. mexicana* arise from the integrated antimalarial, immunomodulatory, and antioxidant properties of the plant. The connecting lines represent proposed functional interactions based on the experimental findings.

Based on the present findings, we propose that the protective effects of the suspension of powdered aerial parts of *A. mexicana* result from the integrated action of its antimalarial, immunomodulatory, and antioxidant activities rather than from a single mechanism (Figure 9). Specifically, the reduction in parasite burden; preservation of CD4^+^ and CD8^+^ T-cell populations; modulation of IL-10, IFN-γ, and IL-17A production; and increased GPx activity may collectively contribute to the maintenance of immune and redox homeostasis during infection. Although this proposed mechanism requires further experimental confirmation, it provides a conceptual framework for future mechanistic studies.

It is important to note that the antimalarial, immunomodulatory and antioxidant activities of a powdered aerial parts suspension of *A. mexicana* are unlikely to depend solely on artemisinin but rather on the combined action of multiple bioactive constituents. Several compounds, including sesquiterpene lactones and volatile terpenoids, are chemically unstable and may undergo degradation during extraction, purification, or storage [46]. Consequently, administration of the powdered aerial-part suspension preserves a broader spectrum of bioactive metabolites, allowing potential synergistic interactions that may enhance the overall pharmacological activity of the plant. Further dose-ranging and pharmacokinetic studies will be necessary to determine the minimum effective dose, characterise exposure to individual constituents, and evaluate the translational potential of *A. mexicana* preparations.

## 4. Materials and Methods

### 4.1. Plant Collection and Treatment Preparation

The *A. mexicana* plants were collected 1 km southeast of San Pablo Ixayoac, Texcoco (19°28′23″ N, 98°47′43″ W). The plants were identified by Dr Eloy Solano-Camacho, and a voucher sample was deposited in the herbarium of the FES Zaragoza, UNAM, under accession number FEZA 16107. The aerial parts were subsequently washed with distilled water and dried at room temperature in the dark for 10 days to prevent the photodegradation of bioactive compounds. The dried material was ground and sieved to obtain a uniform particle size of approximately 0.3 mm; the plant powder was stored in airtight containers protected from light until use. For administration, the suspension of powdered aerial parts of *A. mexicana* was freshly suspended in a 0.5% (*w*/*v*) sodium carboxymethylcellulose solution (CMC-Na) (Sigma-Aldrich, St Louis, MO, USA) immediately before administration. The suspension was administered orally by gavage at a dosage of 1600 mg/kg body weight (200 μL/mouse) once daily for 7 consecutive days.

### 4.2. Experimental Animals

Given that males develop the most severe clinical symptoms, this study focused on CBA/Ca male mice. Animals aged 6–8 weeks old (n = 5) were used; the founding breeding colonies were generously provided by Dr William Jarra (National Institute for Medical Research, Mill Hill, London). The mice were maintained, bred, and housed at the animal facility at FES Zaragoza Universidad Nacional Autónoma de México (UNAM) under strictly controlled conditions. These included filtered air and water, a balanced diet, and sterilised bedding and cages, all within a 12 h light/dark cycle. All experimental protocols were reviewed and approved by the Institutional Ethics Committee on the Care and Use of Animals at FES Zaragoza, UNAM (Registration No. FESZ/CEI/2/0/09/23). All experimental procedures complied with the official Mexican regulatory standard NOM-94062-ZOO-1999, for the care and handling of laboratory animals.

### 4.3. Parasite and Inoculum Preparation

*P. berghei* ANKA was provided by Dr William Jarra (NIMR, London, UK) and was maintained via cryopreservation in liquid nitrogen. To reactivate the parasite, a frozen stabilate was thawed and promptly injected into a four-week-old male mouse. Once parasitemia reached 20%, blood was harvested using phosphate-buffered saline (PBS)/heparin. The total erythrocyte count was determined using a Neubauer chamber, while the percentage of infected cells was assessed in Giemsa-stained blood smears. The blood was diluted in PBS to obtain a final inoculum concentration of 1 × 10^3^ parasitised red blood cells/100 µL, which was administered intravenously to each mouse.

### 4.4. Experimental Design

Two independent experiments were conducted to evaluate the effects of *A. mexicana* in a murine model of experimental malaria.

In the first experiment, a four-day suppressive test was performed to identify the optimal antimalarial dose of *A. mexicana*. Six groups of male mice (n = 5) were infected intravenously on day 0 with 100 μL containing 1 × 10^3^ parasitised erythrocytes of *P. berghei* ANKA. Two hours after infection, the mice were treated orally with vehicle, CLQ, or the suspension of powdered aerial parts of *A. mexicana* at doses of 400, 800, 1600, or 3200 mg/kg, suspended in 0.5% Sodium Carboxymethyl Cellulose (CMC-Na) (Sigma-Aldrich, St. Louis, MO, USA) solution. Treatments were administered daily for four consecutive days (days 0–3 post-infection) following the four-day suppressive test for the evaluation of candidate antimalarial compounds [47]. Parasitemia was monitored daily beginning on day 3 post-infection. Clinical conditions of the animals and survival were monitored daily and recorded from day 0 to day 8 post-infection, which corresponded to the peak of parasitemia and the day the mice were euthanised; no further measurements were taken because, in our experience, the mice in the vehicle-treated group experienced seizures and died after that day [48].

After determining the optimal antimalarial dose, a second independent experiment was conducted to evaluate the immunological and oxidative stress parameters. Because parasitemia suppression declined by day 7 post-infection, presumably because of the rapid metabolism of the plant-derived bioactive compounds, the treatment regimen was extended to seven consecutive days in the second experiment. Six groups of mice (n = 5 per group) were included, comprising three uninfected and three infected groups. Within each infection status, the mice were treated with either vehicle, CLQ, or a suspension of powdered aerial parts of *A. mexicana* (1600 mg/kg) suspended as described above. Parasitemia was monitored daily from day 3 post-infection, while body weight, haemoglobin concentration, and body temperature were recorded throughout the study. On day 8 post-infection, the animals were euthanised, and brain, spleen, and plasma samples were collected for the assessment of oxidative stress biomarkers and immunological parameters.

### 4.5. Assessment of Parasitemia

To evaluate the parasite load, blood smears were fixed in methanol and stained with Giemsa (Sigma-Aldrich). Microscopic analysis was conducted using a Carl Zeiss Standard 20 optical microscope (Carl Zeiss Ltd., Welwyn Garden City, UK). Evaluation was performed using a 100 × oil objective, quantifying infected erythrocytes across 50 distinct fields per sample when parasitemia was under 1%. Data are reported as the geometric mean ± the standard error of the mean (SEM). Each group consisted of five subjects (n = 5) and was evaluated across two independent replicates. To quantify parasite suppression (PSP), the following formula was used:

PSP = [(A − B)/A] × 100

where A corresponds to the geometric mean of parasitemia in the group administered vehicle (VEH), and B is the percentage of parasitemia in each group analysed.

### 4.6. Assessment of Body Temperature

Body temperature was measured daily starting from the day of infection (day 0) through day 8. This was measured using a Thermofocus^®^ infrared thermometer (01500A/H1N1, Vedano Olona-Varese, Italy). The procedure involved aiming the device’s beam at the abdominal area from approximately 5 cm. To ensure data consistency and reduce external interference, all the readings were taken under strictly controlled environmental conditions.

### 4.7. Assessment of Body Weight

Animal body weights were monitored daily from day 0 through day 8 following infection using an Ohaus semianalytical balance (Parsippany, NJ, USA). To track physical condition, weight fluctuations were determined as a percentage of the baseline weight recorded on day 0. These findings are reported as the mean ± SEM for every group.

### 4.8. Determination of Haemoglobin Levels

To assess haemoglobin levels, blood was collected by making a minor incision (approximately 0.5 mm) in the tail of each mouse. Two microliters of blood were sampled and immediately diluted in 498 µL of Drabkin’s reagent (Sigma-Aldrich). The resulting mixture was analysed using a spectrophotometer (Multiskan GO, Thermo Fisher Scientific, Waltham, MA, USA) to measure absorbance at 540 nm. The final concentrations were then determined by comparing these readings against a commercial haemoglobin standard (Sigma-Aldrich).

### 4.9. Splenic Index

On day 8 post-infection, the mice were weighed using an electronic balance (Ohaus), euthanised, and their spleens were excised and weighed on an analytical balance (Sartorius, Göttingen, Germany). The splenic index was determined as the ratio of the spleen weight to the body mass of each animal.

### 4.10. Quantification of Biochemical Markers of Hepatic and Renal Function

To assess hepatic and renal function, plasma concentrations of total protein (TP), albumin (ALB), globulin (GLO), total bilirubin (TBIL), alanine aminotransferase (ALT), aspartate aminotransferase (AST), gamma-glutamyl transferase (GGT), urea, and creatinine were quantified. Briefly, 100 µL of plasma obtained from uninfected mice treated with vehicle or *A. mexicana* were analysed using the MNCHIP Liver and Renal Function Lyophilised Kit (Tianjin, China) according to the manufacturer’s instructions. Measurements were performed using the MNCHIP CelerCare M5 analyzer (Tianjin MNCHIP Technologies Co., Ltd., Tianjin, China).

### 4.11. Quantification of Plasma Cytokines

On day 8 post-infection, the animals were euthanised to facilitate cardiac blood collection into heparinized tubes. The plasma was then isolated and stored at −70 °C until analysis. To quantify the levels of TNF-α, IL-6, IFN-γ, IL-17A, and IL-10, a commercial cytometric bead array (CBA) kit (BS Mouse Th1/Th2/Th17 CBA Kit, BD Biosciences-Pharmingen, Heidelberg, Germany) was used according to the manufacturer’s instructions. The assay utilised a standard curve with a detection limit for each cytokine of: 0.9 pg/mL (TNF-α), 1.4 pg/mL (IL-6), 0.5 pg/mL (IFN-γ), 0.8 pg/mL (IL-17A) and 16.8 pg/mL (IL-10); at 0.625 pg/mL, the average sensitivity was 0.9 ± 0.05 pg/mL, and the interassay variability was 5%.

### 4.12. Analysis of Splenic Cell Populations

Splenic immune cells were evaluated using flow cytometry as previously described [24]. Briefly, on day 8 post-infection, the spleens were harvested under sterile conditions and mechanically dissociated through nylon meshes to create a cell suspension. To isolate the leukocytes, red blood cells were eliminated using commercial lysis buffer (BD Biosciences, San Jose, CA, USA). The remaining cells were washed with PBS, 1% albumin, and 0.1% NaN_3_ (Sigma-Aldrich), counted, and adjusted in a Neubauer chamber to obtain 1 × 10^7^ cells/mL. Each sample was divided into three aliquots of 100 μL; each aliquot (1 × 10^6^ cells) was stained with a previously calibrated mixture for 30 min. The first staining mixture identified T cells (CD3^+^, CD4^+^, and CD8^+^); the second mixture identified B cells and macrophages (CD19^+^ and CD107b^+^); and the third mixture was used to identify NK cells (CD3^−^CD16^+^/32^+^). A total of 10,000 events were acquired for each aliquot, and the doublets and singlets were removed in an FSC vs. SSC plot; then, the uniform region corresponding to the cells of interest was selected. For mix one, SSC vs. APC-CD4^+^ and SSC vs. PE-CD8^+^ were used. The second mixture was used to identify the SSC vs. APC-CD19 dot plot, and CD07b^+^ cells were identified from this region using a dot blot of the SSC vs. PE-antiCD107b^+^. The third mixture was prepared to identify the selected NK cells by plotting the SSC against FITC-CD3^−^ and PE-CD16^+^/32^+^ cells. All antibodies were purchased from BioLegend Global Headquarters, (BioLegend Way, San Diego, CA, USA).

Data were acquired on a FACSAria II flow cytometer (BD Biosciences), and 10,000 events per sample were collected. Gating strategies based on size (FSC), complexity (SSC), and fluorescence intensity were applied using FlowJo software v11 (Tree Star Inc., Ashland, OR, USA).

### 4.13. Determination of P. berghei ANKA-Specific Antibodies

To quantify the humoral immune response, specific IgG and IgM antibodies against *P. berghei* ANKA were measured with an enzyme-linked immunosorbent assay (ELISA) following established protocols [49]. Briefly, 96-well plates were coated with 100 µL of parasite antigen (10 µg/mL) in carbonate buffer and incubated overnight at 4 °C. Nonspecifically bound sites were blocked with 3% skim milk in PBS for 2 h at 37 °C. The plasma samples were diluted 1:20 in PBS containing 0.02% skim milk, added (100 µL/well), and incubated for 1 h at 37 °C. After being washed, the plates were incubated with biotin-conjugated anti-IgG or IgM monoclonal antibodies (Zyme, San Francisco, CA, USA) followed by a streptavidin–peroxidase solution. The enzymatic reaction was developed using O-phenylenediamine (0.4 mg/mL) in citrate buffer (pH 5.0) and 0.03% H_2_O_2_ for 20 min. The absorbance was measured at 492 nm using a Multiskan GO plate reader (Thermo Fisher). Owing to the lack of a known concentration standard, the results are reported in absorbance units. Values were compared against an internal reference plasma pool obtained from eight-week-old naïve CBA mice (n = 5).

### 4.14. Quantification of the Antioxidant Activities of the Enzymes Superoxide Dismutase (SOD), Glutathione Peroxidase (GPx) and Catalase (CAT)

To evaluate oxidative stress markers, the specific activities of SOD, GPx, and CAT were quantified in tissue homogenates. On day 8 post-infection, the animals were euthanised, and the spleen and brain were harvested and rinsed with chilled PBS. The tissues were mechanically dissociated in 1 mL of sterile PBS and stored frozen at −70 °C until analysis in the presence of BHT (butylated hydroxytoluene; Sigma-Aldrich).

#### 4.14.1. SOD

SOD activity was determined using the RANSOD method (Randox Laboratories, Antrim, UK). Spleen and brain suspensions were diluted 25-fold in PBS (pH 7.0), and a 25 µL aliquot was reacted with the xanthine oxidase substrate. The reaction kinetics were monitored at 505 nm using a Multiskan GO spectrophotometer (Thermo Fisher Scientific). The results are expressed as units per milligram of protein (U/mg protein).

#### 4.14.2. GPx

GPx activity was measured using RANSEL reagents (Randox Laboratories). Spleen and brain suspensions (50 µL) were diluted according to the manufacturer’s protocol. The specific activity was determined by measuring the rate of decrease in absorbance at 340 nm, reflecting the oxidation of NADPH. The data are reported as U/mg protein.

#### 4.14.3. CAT

CAT activity was assessed using a previously described method [34]. Spleen and brain tissues were diluted 1:500 in PBS. A 1 µL aliquot of the sample was added to 500 µL of 30 mM hydrogen peroxide (H_2_O_2_). The absorbance was recorded at 240 nm at two time points: immediately (A1) and after 1 min (A2). The reduction in absorbance represents the amount of H_2_O_2_ consumed per minute per mg of protein.

### 4.15. Quantification of Malondialdehyde (MDA) Concentrations in the Spleen and Brain

To evaluate lipid peroxidation in the spleen and brain, MDA levels were quantified using the thiobarbituric acid reactive substances (TBARS) method, as described above [50]. Briefly, tissue homogenates (standardised to 1 mg of protein) or MDA standards (1,1,3,3-tetramethoxypropane (Sigma-Aldrich)) were combined with 100 µL of 0.2 M orthophosphoric acid (Sigma-Aldrich), 12.5 µL of 2 mmol/L BHT (butylated hydroxytoluene (Sigma-Aldrich)) and 12.5 µL of 0.1 M thiobarbituric acid (TBA) (Fluka Chem, Buch, Switzerland (Sigma-Aldrich)) solution (0.1 M TBA in 0.11 M NaOH). The mixture was incubated at 90 °C for 45 min to promote the formation of MDA-TBA adducts and then promptly cooled on ice to stop the reaction. For chromogen extraction, 250 µL of n-butanol was added, and the samples were shaken to create an emulsion. The organic phase was isolated by centrifugation at 1500× *g* for 3 min. The absorbance was measured at 535 nm using a Multiskan GO microplate reader (Thermo Fisher Scientific). The final MDA concentrations were determined by interpolation from the standard curve.

### 4.16. Determination of Nitrite/Nitrate Levels as an Indicator of Nitric Oxide Production

Nitrite and nitrate concentrations were measured as indirect indicators of nitric oxide (NO) production using the Griess reaction, as previously described [45], with minor modifications. Briefly, spleen, brain, and plasma homogenates (1 mg protein) were incubated with nitrate reductase and NADPH to convert nitrate to nitrite. Griess reagent was then added, the proteins were precipitated with trichloroacetic acid, and the supernatant was collected after centrifugation. The absorbance was measured at 540 nm using a Multiskan Go microplate reader (Thermo Scientific, Vantaa, Finland).

### 4.17. Phytochemical Characterisation of the Filtrate Obtained from the Suspension of Powdered Aerial Plant

#### 4.17.1. Preparation of the HPLC Sample from the Suspension of the Powdered Aerial Plant

The aerial parts of *A. mexicana* were subsequently washed with distilled water and dried at room temperature in the absence of direct light for 10 days to minimise the photodegradation of bioactive compounds. The dried material was ground and sieved to obtain a homogeneous particle size of approximately 0.3 mm. A suspension of powdered aerial parts of *A. mexicana* was prepared in 0.5% CMC.

To chemically characterise the pharmacological preparation administered to the animals, an HPLC sample was prepared from the same plant suspension described in Section 4.1. Briefly, 1.5 g of dried plant material was suspended in 12 mL of drinking water containing 0.5% carboxymethyl cellulose (CMC), using the same formulation as that administered to the animals. The mixture was allowed to stand at room temperature for 30 min and then filtered under reduced pressure to remove particulate matter prior to chromatographic analysis. The resulting filtrate was diluted 1:100 with water to avoid detector saturation. The artemisinin concentration was determined using an external calibration curve prepared from a commercial artemisinin standard.

#### 4.17.2. HPLC-DAD Conditions

Chromatographic analyses were performed using a Hewlett Packard Series 1100 HPLC system equipped with a diode-array detector (DAD/PDA). Separation was achieved on a Nucleodex β-OH column (Macherey-Nagel, Düren, Germany, 200 × 4 mm, 5 µm). An isocratic mobile phase consisting of water (82%, solvent A), acetonitrile (9%, solvent B), and methanol (9%, solvent C) was used. Prior to each analysis, the column was equilibrated with solvent A for 5 min. The flow rate was maintained at 0.6 mL/min, the autosampler temperature was set at 20 °C, and chromatograms were acquired at 210 nm.

#### 4.17.3. Artemisinin Quantification

An artemisinin standard (Sigma-Aldrich, Lot # ENC115, 98% purity) was used as the external standard for quantification. The initial concentration of the filtered sample and the artemisinin standard was 1000 μg/mL. The injection volume was 20 µL. Four independent injections were performed. An external calibration curve was constructed using seven concentrations of artemisinin standards (20, 10, 5, 2.5, 1.25, 0.60, and 0.30 µg). Concentrations are expressed in artemisinin mg/g dried powdered aerial parts of *A. mexicana*.

### 4.18. Detection of Plasmodium berghei ANKA DNA in Brain Tissue

Parasite DNA in the brains of infected mice was detected by nested PCR using two previously described primer pairs [43]. PCR products were separated on a 10% polyacrylamide/bis-acrylamide gel, stained with ethidium bromide, and visualised using a gel documentation system (Kodak/Sigma T1202, Rochester, NY, USA). The expected amplicon size was 865 bp, and the band intensity was quantified by densitometric analysis.

### 4.19. Statistical Analysis

Statistical analyses were performed using GraphPad Prism (version 9.5.0). First, normality was assessed using Levene’s test at the 95% confidence level. Differences between groups were subsequently evaluated using one-way analysis of variance (ANOVA) followed by Bonferroni’s multiple-comparison test. Temporal changes in physiological parameters (body temperature, haemoglobin concentration, and body weight) from day 0 to day 8 post-infection were summarised using the area under the curve (AUC) method. The percentage of parasite suppression was calculated for the dose–response experiments to determine the optimal therapeutic dose. Each experiment was analysed independently, *p* < 0.05 was considered statistically significant.

## 5. Conclusions

Collectively, these findings demonstrate that treatment with the suspension of powdered aerial parts of *A. mexicana* has antimalarial, immunomodulatory, and antioxidant effects on experimental malaria caused by *Plasmodium berghei* ANKA. The selected dose reduced parasitemia and preserved physiological parameters associated with disease severity, including haemoglobin concentration and body weight. Treatment also modulated splenic immune cell populations and cytokine production, while enhancing glutathione peroxidase activity, with no evidence of increased lipid peroxidation or significant alterations in nitric oxide metabolites under the experimental conditions evaluated. These findings support further investigations of the bioactive compounds present in this pharmacological preparation, their mechanisms of action, and their potential development as adjunctive therapies for malaria.

## Figures and Tables

**Table 1:** Major phytochemicals previously reported in *A. mexicana* and their identification in this study.

Compound	Reference	Status in the Present Study
Artemisin	Present study; [14]	Confirmed by HPLC-DAD
Estafiatin	[15,16]	Not identified
Achillin	[17]	Not identified
Eupatilin	[15,16,18]	Not identified
Artemetin	[19]	Not identified
Jaceosidin	[20]	Not identified
Salvinive	[20]	Not identified

**Table 2:** Antimalarial activity of *A. mexicana* against *P. berghei* ANKA on days 5 and 7 post-infection.

Dose (mg/kg)	Geometric Mean Parasitemia (% ± SD) Day 5	Parasitemia Suppression (%) Day 5	Geometric Mean Parasitemia (% ± SD) Day 7	Parasitemia Suppression (%) Day 7
**VEH**	2.11 ± 0.75	----------	5.38 ± 0.41	-----------
**CLQ 25**	0.89 ± 0.41	57.79	1.86 ± 0.79	65.40
**ART 400**	0.94 ± 0.37	55.07	4.79 ± 1.03	11.0
**ART 800**	0.70 ± 0.67	66.66	6.06 ± 1.35	−12.53
**ART 1600**	**0.59 ± 0.28**	**72.02**	**3.66 ± 0.57**	**31.96**
**ART 3200**	2.04 ± 0.54	30.03	5.29 ± 1.5	1.73

VEH (vehicle), CLQ (chloroquine), ART (suspension of powdered aerial parts of *A. mexicana*), (n = 5).

**Table 3:** Serum biochemical parameters in mice treated with *A. mexicana*.

Parameter	Vehicle	*A. mexicana* 1600 mg/kg	Reference Interval	Units	Reference
Total protein (TP)	35.8 ± 5.1	33.3 ± 2	48–53.3	g/L	[21]
Albumin (ALB)	17.6 ± 5.4	17.2 ± 0.9	25.2–28	g/L	[21]
Globulin (GLO)	18 ± 0.8	16.1 ± 1.2	23.3 ± 0.6	g/L	[22]
Total bilirubin (TBIL)	2.7 ± 1.1	2.6 ± 0.5	1.37 ± 0.17	μmol/L	[22]
ALT	≤5	≤5	24–40	U/L	[21]
AST	≤5	9.75 ± 5.6	40–60	U/L	[21]
GGT	≤5	≤5	6.9	U/L	[23]
Urea	5.37 ± 0.9	3.8 ± 0.3	9.86–12.05	mmol/L	[21]
Creatinine	≤20	≤20	10.2	μmol/L	[22]

Data are expressed as mean ± SD. Values at or below the lower analytical measurement limit are indicated by “≤”. Statistical comparisons were performed using the Mann–Whitney U test. No statistically significant differences were detected between the vehicle- and *A. mexicana*-treated groups for the analysed parameters (*p* > 0.05). ALT (alanine aminotransferase), AST (aspartate aminotransferase), and GGT (gamma-glutamyl transferase).

## Data Availability

The original contributions presented in this study are included in the article/Supplementary Materials. Further inquiries can be directed to the corresponding author.

## Supplementary Materials

The following supporting information can be downloaded at: https://www.mdpi.com/article/10.3390/ijms27177637/s1.

## Abbreviations

The following abbreviations are used in this manuscript:

ALB	Albumin
ALT	Alanine aminotransferase
AST	Aspartate aminotransferase
ART	Suspension of powdered aerial parts of *Artemisia mexicana*
AUC	Area under the curve
CAT	Catalase
CD	Cluster of differentiation
CLQ	Chloroquine
ELISA	Enzyme-linked immunosorbent assay
GGT	Gamma glutamyl transferase
GLO	Globulin
GPx	Glutathione peroxidase
IFN-γ	Interferon gamma
Ig	Immunoglobulin
IL	Interleukin
iNOS	Inducible nitric oxide synthase
MDA	Malondialdehyde
NF-κB	Nuclear factor kappa B
NK	Natural killer cell
PBS	Phosphate-buffered saline
*P. berghei* ANKA	*Plasmodium berghei* ANKA
ROS	Reactive oxygen species
SOD	Superoxide dismutase
TBIL	Total bilirubin
Th	T helper cells
TNF-α	Tumour necrosis factor alpha
TP	Total protein
VEH	Vehicle
WHO	World Health Organisation
